# A Digital App-Based Intervention (Platoniq) to Reduce Loneliness: Single-Arm Pilot Study

**DOI:** 10.2196/99374

**Published:** 2026-10-22

**Authors:** Silvan Hornstein, Jasmina Schmidt, Lara Sachs, Tobias Krieger, Daniel Stachnik, Ulrike Lueken

**Affiliations:** 1 Department of Psychology, Humboldt-Universität zu Berlin, Rudower Chausee 18, Berlin, 12489, Germany, 49 15753685796; 2 SHS Social Health Solutions UG (haftungsbeschränkt), Berlin, Germany; 3 Department of Clinical Psychology and Psychotherapy, Institute of Psychology, University of Bern, Bern, Switzerland; 4 German Center for Mental Health (DZPG), Partner site Berlin-Potsdam, Berlin, Germany

**Keywords:** loneliness, social health, digital health, pilot study, digital mental health

## Abstract

This single-arm pilot study of a psychological, app-based intervention (platoniq) found significantly reduced loneliness after 4 and 8 weeks.

## Introduction

Loneliness, the subjectively perceived lack of social relationships, is affecting a significant share of people across countries and age groups [1]. As this is associated with numerous adverse health consequences [2], effective interventions to tackle loneliness are highly needed. Although a substantial body of evidence has examined such interventions and found that psychological approaches in particular can effectively reduce loneliness [3], considerably more research is still required. Loneliness is linked to hypervigilance and negative social cognition, reinforcing withdrawal in a cycle [4], which cognitive-behavioral techniques such as social skills training and cognitive restructuring aim to break. Particularly, low-threshold digital interventions are an interesting yet underinvestigated approach with few available studies [5]. Consequently, the objective of this preregistered [6] pilot study was the evaluation of a digital app-based intervention against loneliness.

## Methods

### Ethical Considerations

Ethical approval for this study was granted by the ethical committee of the Institute for Psychology of the Humboldt University Berlin (identifier: 2025‐58).

### Study Design

This pilot study was designed as a one-arm, noncontrolled trial. Loneliness, the primary outcome, was assessed using the 9-item version of the UCLA Loneliness Scale (UCLA-9) [7] at baseline, 4 weeks, and 8 weeks. Additionally, perceived social support [8], perceived stress [9], and social competencies [10] were measured at baseline and at week 8 as secondary outcomes.

### Sample

Study participants were recruited through convenience sampling via several channels, such as social media and university newsletters. A total of 72 participants matched our inclusion criteria (UCLA-9 score ≥18, reflecting preexisting low-to-moderate loneliness; ≥18 years old; German-speaking; no acute mental health crisis; and not currently seeking treatment), completed at least 1 of the follow-up questionnaires, and accessed the intervention with their phone. The median age was 35 (IQR 27.0-48.75; mean 39.3, SD 14.23) years and 60 (83.3%) participants were female. Of the 72 participants, 10 (13.9%) reported a migration background; 40 (55.6%) were employed full-time; and the sample was highly educated, with the majority (n=50, 69.4%) holding a university degree.

### Intervention

The app-based intervention platoniq (SHS Social Health Solutions UG) was used in this study. The digital intervention is structured into 6 modules, addressing diverse aspects of social relationships and loneliness. Exercises are adapted from social skills training and cognitive behavioral therapy, following a cognitive model of loneliness [4]. One new module is unlocked each week, covering the following topics in sequence: initiating relationships, deepening relationships, emotional support, conflict management, setting boundaries, and social relationships and own needs. Within each module, exercises have to be completed in sequential order (see Figure 1). In addition, the app includes a tracking mechanism for social relationships, allowing participants to monitor their social interactions over the course of the program. Notifications reminded participants to use the app, and chat support was available for technical questions.

**Figure 1.**
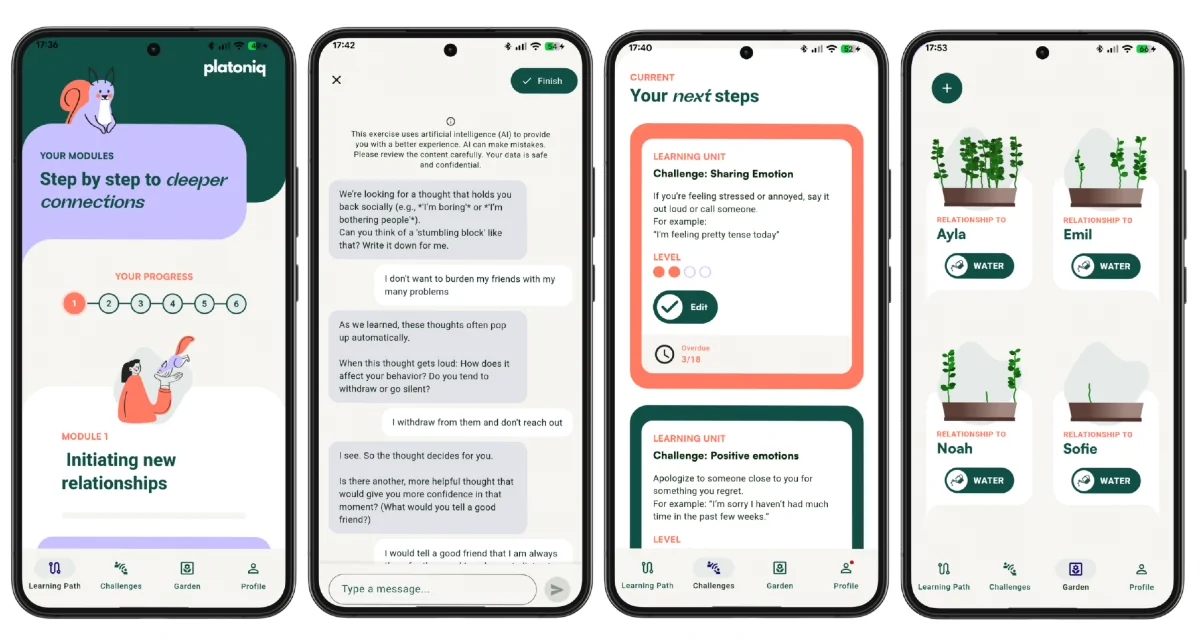
Screenshots of the intervention app, platoniq, which includes exercises (eg, social skills training and cognitive restructuring), behavioral experiments, and a tracking mechanism.

## Results

Out of the 72 included participants, 67 (93%) provided answers to the UCLA-9 after 4 weeks from the intervention start. UCLA-9 scores decreased from baseline (mean 25.6, SD 3.7) to week 4 (mean 23.5, SD 4.2). A paired *t* test showed a significant medium-to-large effect for loneliness (*t*_66_=5.8; *P*<.001; *d*=0.71), with an average reported decrease on the UCLA-9 of 2.2 (SD 3.1). Approximately 75% (50/67) of participants reported a decrease, and 45% (30/67) of all participants reported a decrease of at least 3 points. This has been considered clinically meaningful in past work, following a reliable change index derived for the UCLA-9 in a comparable sample [4]. Higher baseline UCLA-9 scores were associated with greater reduction (*r*=0.25; *P*=.04). The effect remained stable for the 44 participants who responded to the questionnaires after 8 weeks, with scores decreasing from baseline (mean 26.2, SD 4.2) to week 8 (mean 24.0, SD 5.2*; t*_43_=3.8; *P*<.001; *d*=0.57), reporting an average reduction of 2.21 (SD 3.9). Additionally, effects were found for interpersonal competencies (*t*_43_=−3.1; *P*=.004; *d*=−0.46) as well as perceived social support (*t*_43_=−2.4; *P*=.02; *d*=−0.37). However, while there was an average decrease of 0.77 (SD 6.8) in perceived stress, this effect was not statistically significant (*t*_43_=0.75; *P*=0.45; *d*=0.13). No significant baseline differences were found between completers and noncompleters (ie, no 8-week questionnaire) at 8 weeks across demographic or clinical variables, suggesting limited attrition bias. Noncompleters showed markedly lower overall engagement (mean 14.8, SD 13.3 vs 30.9, SD 20.1 events; *t*_69.87_=4.10; *P*<.001).

On average, the included participants used the app for approximately 128 (SD 119) minutes, corresponding to a weekly usage of around 21 (SD 19.8) minutes. On average, 25 (SD 19.3) exercises were completed, as well as 6 (SD 4.6) behavioral experiments, and the tracking mechanism was used 18 (SD 19.3) times. The small (*r*=0.14) correlation between usage time and reduction was nonsignificant in the given sample (*P*=.24).

## Discussion

In this uncontrolled pilot study, an app-based digital health intervention targeting loneliness in individuals with at least low-to-moderate levels of loneliness showed preliminary signals of reduced loneliness. The observed within-group effect size (*d*=0.71) falls within the range reported across existing internet-based interventions (*d*=0.33 to *d*=1.47) [5], corroborating the general promise of this intervention format. However, given the single-arm design, this interpretation is limited, and randomized controlled trials are needed to confirm the intervention’s effectiveness.

The sample was predominantly female and highly educated, with considerable dropout, limiting generalizability. Additionally, user satisfaction and barriers to use were not systematically assessed. Finally, the association between baseline severity and reduction may partly reflect regression to the mean. Interestingly, contrary to our initial hypothesis, the effect on perceived stress in this study was not significant. This may be due to limited statistical power to detect small effects.

Taken together, these preliminary results suggest app-based cognitive behavior therapy interventions may offer a promising approach to addressing loneliness as a pressing public health challenge.

## Abbreviations

UCLA-9: 9-item version of the UCLA Loneliness Scale
